# Effect of concurrent training in unilateral transtibial amputees using Paralympic athletes as a control group

**DOI:** 10.1016/j.clinsp.2023.100165

**Published:** 2023-04-08

**Authors:** Marcus Vinicius Grecco, Guilherme Carlos Brech, Jose Maria Soares-Junior, Edmund Chada Baracat, Júlia Maria D'Andrea Greve, Paulo Roberto Santos Silva

**Affiliations:** a Laboratory Study of Movement, Instituto de Ortopedia e Traumatologia do Hospital das Clínicas (IOT-HC) da Faculdade de Medicina da Universidade de São Paulo (FMUSP), São Paulo, SP, Brazil; b Sports Medicine Division, Instituto de Ortopedia e Traumatologia do Hospital das Clínicas (IOT-HC) da Faculdade de Medicina, Universidade de São Paulo, São Paulo, SP, Brazil; c FIFA Medical Centre of Excellence, São Paulo, SP, Brazil; d Graduate Program in Aging Sciences, Universidade São Judas Tadeu (USJT), São Paulo, SP, Brazil; e Departamento de Obstetrícia e Ginecologia, Disciplina de Ginecologia, Hospital das Clínicas da Faculdade de Medicina da Universidade de São Paulo (FMUSP), São Paulo, SP, Brazil

**Keywords:** Transtibial Amputation, Concurrent Training, Rehabilitation, Ergospirometry

## Abstract

**Context:**

Transtibial Amputation (TA) predisposes to a sedentary lifestyle.

**Objectives:**

To evaluate the efficiency of a short-term (8-week) Concurrent Training (CT) program in Unilateral Transtibial Amputees (UTA) and to compare it with the physical condition of a group of Paralympic athletes in preparation for the Rio de Janeiro Paralympics.

**Design:**

This was a longitudinal, prospective and controlled trial study.

**Methods:**

Thirty-four male subjects with UTA and using prostheses for six months or more were selected for this study. They were divided into two groups: Group 1 (G1) ‒ 17 non-athlete and untrained UTA and Group 2 (G2) ‒ 17 paralympic athletes with active UTA in the training phase. G1 was evaluated before and after eight weeks of CT and G2 made a single evaluation for control. All were submitted to anamnesis, clinical evaluation (blood pressure, electrocardiogram, and heart rate) and cardiopulmonary exercise testing on a lower limb cycle ergometer, and isokinetic knee dynamometry. The CT of G1 included resistance exercise and aerobic interval training on a stationary bicycle and G2 followed the training of the Paralympic teams.

**Results:**

Patients were retested by the same methods after CT. The two most important central dependent variables (maximal oxygen uptake and muscular strength) increased by 22% and knee extensor and flexor strength by 106% and 97%, respectively.

**Conclusion:**

After eight weeks of CT, there was an improvement in general functional condition, muscle strength, and cardiorespiratory performance improving protection against chronic diseases and quality of life.

## Highlights

• Unilateral transtibial amputees and functional deficits.
• Concurrent training proved to be effective.
• Paralympic athletes should review their physical training.

## Introduction

Transtibial Amputation (TA) is considered a great level of amputation, favoring rehabilitation and fitting. TA affects men (75% of cases) and the main cause in the population aged between 50 and 75 years is vascular, but in the young population, traumatic amputations related to work and traffic accidents predominate.^1^ TA predisposes to a sedentary lifestyle due to gait difficulties and can lead to sarcopenia, changes in postural balance, osteoporosis, and predispose to metabolic syndrome.^2^^,^^3^ TA gait increases heart rate and oxygen consumption (VO_2_) by 5% compared to non-amputees. Resistance exercises for lower limbs are essential for TA, as they improve prosthesis loading and reduce energy expenditure.^3^

Several patients with TA, even after the prosthesis and rehabilitation programs, do not reach a great functional performance, presenting an unfavorable physical condition for the daily use of the prosthesis, evidencing the need for a structured physical conditioning program to improve functional performance.^4^ Due to this condition, maintaining gait quality and functional independence of the UTA requires muscle strength, agility, balance, postural control, and great aerobic condition.^5^ Resistance exercises improve the condition of the stump, improving balance and better use of the prosthesis and the improvement of the functional condition contributes to the prevention of diseases related to a sedentary lifestyle.^5^ The literature shows that an increase of 1 MET, corresponding to 3.5 mL/kg/min in VO_2_max, is associated with a 13% and 15% lower risk of all-cause mortality and cardiovascular events, respectively.^6^ A sedentary lifestyle and excessive energy expenditure, 16% more energy for walking, place transtibial amputees in a cardiovascular risk zone.^7^^,^^8^

Sports activities play an important role in the social integration of amputees and many become Paralympic athletes within the modalities available for this type of physical disability. Some sports are practiced without the prosthesis and others, such as running, with the use of the prosthesis. The sports practiced by the UTA in this study were: sitting volleyball, athletics (running), and swimming. Specific training for each sport sought to develop muscle strength, balance, agility, technical capacity for each sport, and cardiorespiratory condition.

The functional demand of the Paralympic athlete with UTA is similar to athletes without disabilities, especially runners. Therefore, physical condition training must provide strength gain (including the amputated limb) and cardiorespiratory condition, in addition to special care for balance gain and weight distribution between limbs for adequate performance.

In contrast to experimental settings in the real world and for the general population, the combination of resistance exercise and aerobic resistance simultaneously, in the same session (CT), has been considered a training program that leads to superior adaptations in health-related variables and body function, regardless of age or sex, including increases and/or improvements in basal metabolic rates, insulin sensitivity, glucose/lipid metabolism, lipid profile, and body composition, while muscle hypertrophy/strength/power and endurance capabilities are increased. Several studies have provided strong evidence that CT adaptations further increase endurance.^7^ It appears that CT innervations do not negatively affect resistance training adaptations.^8^^,^^9^ These were the reasons adopted for using this training methodology.^10^

Intervention programs to improve the rehabilitation process and the quality of life of amputees is a necessity, as it is a life-changing event associated with poor physical and psychological functioning with decreased quality of life.^11^ Therefore, the aim of this study was to verify the effect of a short-term program on muscle strength, cardiorespiratory fitness, and clinical signs with ATU after eight weeks of concurrent training in relation to Paralympic athletes trained and evaluated six months before the 2016 Paralympics. The hypothesis of the present study suggests that eight weeks of supervised CT (independent variable) are sufficient to improve strength, muscular endurance, and body composition parameters and increase cardiorespiratory fitness (dependent variables) in young patients with ATU after discharge from the rehabilitation program and in regular use of the prosthesis.

## Methods

### Study location and ethical issues

The study was carried out at the Laboratory of Movement Studies (LEM) of the Institute of Orthopedics and Traumatology, Faculty of Medicine, University of São Paulo (IOT-HC-FMUSP). The Ethics Committee for the Analysis of Research Projects case CAAE number 82,388,117.3.0000.0068 approved the study. All subjects received and signed a free and informed ethical consent. The study complied with the Declaration of Helsinki (2013).

### Type of study and participants

It was an interventional, prospective, controlled, cross-sectional, and comparative study. The trial was conducted between December 2013 and February 2016 at the Institute of Orthopedics, Hospital das Clínicas, Faculty of Medicine, University of São Paulo (IOT-HC-FMUSP).

### Sample

Initially, 50 participants with Unilateral Transtibial Amputation (UTA) were evaluated and 34 met the inclusion criteria. The sample was divided into two groups: Group 1 (G1) ‒ 17 patients with unilateral transtibial amputation non-athletes (evaluated before and after training) and Group 2 (G2) ‒ 17 participants with unilateral transtibial amputation, Paralympic athletes in training (classified or in classification phase) for the 2016 Paralympics. G1 patients were evaluated at the beginning and after the 8-week intervention period of training and G2 underwent a single evaluation and should have been in training at the time of evaluation.

The inclusion criteria for G1 were as follows: (i) UTA (any etiology); (ii) Age between 18‒50 years; (iii) Abnormal electrocardiogram; (iv) Absence of the previous injury of any etiology in the last three months before the evaluation; (v) Absence of any other musculoskeletal disease, (vi) Inflammatory, neurological and psychiatric disease; (vii) No smoking and (viii) Use of a prosthesis for three or more months. The inclusion criteria for G2 were as follows: (i) A federated athlete and (ii) A member of the sports teams participating in the 2016 Paralympics in training at the time of the evaluation. In this study, 59% were sitting volleyball athletes, 29% were athletics, and 12% were swimming. A specific type of sport was not an inclusion criterion. Exclusion criteria were: (i) Pain or inability to complete any of the tests; (ii) Arterial hypertension (above 130/90 mmHg) before strength assessment; (iii) Loss of three consecutive training sessions and (iv) Non-attendance of the reassessment session for G1. G1 participants were recruited from the Amputee Ambulatory at IOT-HC-FMUSP and G2 participants were recruited from teams and sports centers specialized in Paralympic sports, which were in the preparation phase for the 2016 Rio de Janeiro Paralympics. That all G2 athletes practiced weight training to assist in their specific sports. The seated volleyball group did a stationary bike to train in aerobics. The aerobic training performed by volleyball athletes lasted from 20 to 30 minutes and had subjective control of loads. Therefore, it was not a metric controlled with scientific rigor. Even weight training, performed by everyone and did not have an evolutionary control of the loads. They performed the exercises according to their sense of well-being. This procedure does not consider the principle of increasing overload to achieve favorable training effects.

### Clinical evaluation

Anamnesis (inclusion criteria), physical examination (blood pressure, Body Mass Index [BMI], thigh, and abdominal circumference). Blood pressure was measured in the sitting position with oscillometric devices according to the 2018 European Society of Cardiology/European Society of Hypertension guidelines.^12^ Arterial hypertension was defined as systolic BP ≥ 140 mmHg and/or diastolic BP ≥ 90 mmHg^13^ or the use of antihypertensive drugs was present. BMI was calculated by dividing body weight in kilograms by the square of body height in meters. Obesity was defined as a BMI ≥ 30 kg/m^2^; overweight, such as a BMI of 25‒29.9 kg/m^2^; and normal weight, such as BMI < 25 kg/m^2^.^12^

### Lower body strength assessment

The isokinetic knee strength was assessed utilizing the Isokinetic dynamometry Biodex® Multi-joint System 3 (Biodex Medical^TM^, Shirley, NY, USA). The isokinetic dynamometer was calibrated thirty minutes before starting the tests. Before the isokinetic testing, patients had a 5-min self-paced warm-up on a computerized braked cycle ergometer (Bike, Cardiovascular Systems, Cybex, USA). After a standardized warm-up, the subjects were positioned for concentric evaluation of extension and flexion movements of the knee joint. They remained seated with the hips at 90° of flexion and secured to the chair by belts. The test started with the dominant limb. The limb was evaluated by positioning the lateral condyle of the femur in alignment with the mechanical axis of the dynamometer. All subjects performed four submaximal repetitions to become familiar with the equipment, followed by a 60-second rest interval, then five maximal repetitions of knee extension and flexion starting with the non-amputated limb. Constant standardized verbal encouragement was given during the tests in order to promote maximum effort during contractions. The isokinetic variables used were maximum Peak Torque Corrected for Body Weight (PTQ/BW) and Total Work (TW).^13^

### Cardiopulmonary exercise testing (CPET)

The patients completed an incremental maximal cardiopulmonary exercise testing until they reached exhaustion on a cycloergometer (Cybex™ cycle ergometer, USA) by an exercise physiologist. The patients were tested utilizing the Astrand modified protocol with an initial load of 25 watts and an increment of 25 watts every two minutes with a constant speed between 60 and 70 rpm. A breath-by-breath analysis was performed on the was measured by a computerized system (CPX-Ultima Medical Graphics™ Metabolic System, St. Paul, Minnesota, USA). Peak oxygen uptake (VO_2_peak), first and second ventilatory thresholds (VT_1_ and VT_2_). VT_1_ was determined when the Ventilatory Oxygen Equivalent (VE/VO_2_) and End-Expiratory Oxygen Pressure (PETO_2_) reached the lowest value, preceded by a continuous increase in both values, associated with an abrupt increase in the respiratory quotient (RQ = VCO_2_/VO_2_). VT_1_ is the first inflection of the curve in progressive exercise and the Respiratory Compensation Point (RCP) for VO_2_. VT_2_ was determined when the Ventilatory Equivalent of Carbon Dioxide (VE/VCO_2_) reached the lowest value preceded by its increase and the End-Expiratory Pressure of Carbon Dioxide (PETCO_2_) reached the highest value preceded by its reduction. VT_2_ is the second inflection of the curve in progressive exercise and the Respiratory Compensation Point (RCP) for VCO_2_.^14^ The variables used for analysis were VO_2_peak, load in watts, O_2_ pulse, Maximum Heart Rate (HRmax), test duration, mechanical efficiency, and percentage of absolute VO_2_ and HRmax. The loss of verbal communication with a subject during the test was overcome with prearranged hand signals. Measurement of peak oxygen uptake (VO_2_peak) and other cardiorespiratory variables was repeated on each individual patient using an identical protocol after eight weeks of supervised physical training. The VO_2_max or VO_2_peak was taken as the highest VO_2_ value obtained in any 10-s period and was stated as being achieved by the following end-point criteria: (a) Oxygen uptake did not increase > 2.1 mL/kg/min (plateau in VO_2_) despite the increasing workload, (b) With Respiratory Exchange Ratio (RER) values > 1.1, (c) Maximal HR within five beats/min of the age-predicted maximum by Tanaka equation (HRmax = 208 - [0.7*age]), (d) More than 18 on subjective Borg's scale.

### Aerobic training design

All transtibial amputees performed two sessions of incremental exhaustive exercise testing, separated by a period of eight weeks. The subjects enrolled in the control group (Paralympic athletes) maintained their usual physical activities and were evaluated only once. Participants in the Experimental Group (G1) underwent eight-week supervised physical training with the prosthesis. The training was performed three times a week and was supervised by a team of specialists in physical conditioning, rehabilitation, and exercise physiology. The model used in the present study was aerobic interval training lasting 20 minutes on a stationary bicycle, following the following steps: three minutes as a low-intensity stimulus in the load (watts) corresponding to VT_1_ and two minutes in the load (watts) corresponding to VT_2_ as a high-intensity stimulus. A heart rate monitor (Polar F1™) was used during aerobic training to control exercise intensity.^14^

### Resistance exercises training

They were performed three times a week. The muscles were trained: (a) Knee extensors and flexors, (b) Ankle plantar flexors, (c) Pectorals, (d) Scapula stabilizers, (e) Shoulder abductors, (f) Lumbar extensors, (g) Abdominals, and (h) Hip adductors. To determine the training load, the test of One-Repetition Maximum (1RM) was used. Three decreasing sets were performed (12/10/8 repetitions) with 60% of 1RM in the upper limbs and 80% in the lower limbs. The non-amputated limb made the same load as the amputee to equalize the forces between the limbs. Loads were adjusted every two weeks following the progression principle.^15^^,^^16^ The resistance exercise training protocol was based on recommendations from the American College of Sports Medicine, American Heart Association, and National Strength and Conditioning Association;^15^^,^^16^ i.e., three sets of 8 to 12 repetitions of 8 to 10 resistance exercises involving major muscle groups (arms, shoulders, abs, back, hips, and legs) on 2 to 3 days a week with multiple sets potentially offering greater improvements.^16^

### Statistical analysis

Data were verified for normality using the Shapiro-Wilk test result. Descriptive statistics are presented as mean ± standard deviation for continuous variables. Independent samples *t*-test or Mann-Whitney *U* test result was used to compare data among groups at baseline, where appropriate. The Rank Sum Test was used to assess the amputation time of the groups. Fisher's exact test was used to compare gender and side of amputation. The *t-*Student test was used to assess Body Mass Index (BMI), age, and all isokinetic, clinical, cardiorespiratory, and dynamometry variables in the comparisons between G1 (pre/post) and G2. Wilcoxon Sign Rank test result was used to compare G1 pre with G1 post in clinical, cardiorespiratory, and strength variables. The size of the training effect of G1 was evaluated by the *d* Cohen index: insignificant (*d* < 0.19), small (*d* = 0.20‒0.49), moderate (*d* = 0.50‒0.79), large (*d* = 0.80‒1.29) and very large (*d* > 1.30). The analysis was performed with the aid of Minitab (version 15) and SPSS (version 18). The significance level for testing the hypotheses was set to 0.05.

## Results

In the whole sample population, the mean age was 32 years, Table 1 presents the physical characteristics of amputees from both groups.

**Table 1:** Baseline physical characteristics of the Group 1 (G1) Unilateral Transtibial Amputation (UTA) patients and Group 2 (G2) – Unilateral Transtibial Amputation (UTA) paralympic athletes (mean and standard deviation).

Parameters	G1 (n = 17)	G2 (n = 17)	p-value
Age (years)	30.5 ± 8.4	32.8 ± 7.9	0.4
BMI (kg/m^2^) (G1 pré vs. G2)	26.7 ± 5	26.8 ± 4	0.9
Amputation time (months)	31.5 ± 44.3	81.2 ± 67.6	0.02^a^
Men	13 (77%)	15 (88%)	0.7
Women	4 (23%)	2 (12%)	0.7
Amputation right side	7 (41%)	13 (77%)	0.08
Amputation left side	10 (59%)	4 (23%)	0.08

a p ≤ 0.05; ** p ≤ 0.01. Tests: *t*-Student for age and BMI (Body Mass Index); Rank Sum for amputation time and Fisher for gender and amputation side.

Of the group studied, 88% of the patients wore a prosthesis for six or more months, 89% were of traumatic origin, 62% were overweight (BMI ≥ 26 kg/m^−2^), 35% were hypertensive, and 53% had increased abdominal circumference with a high risk of developing chronic diseases (Table 2).

**Table 2:** Comparison of clinical variables of the Group 1 (G1) Unilateral Transtibial Amputation (UTA) patients and Group 2 (G2) – Unilateral Transtibial Amputation (UTA) paralympic athletes (mean and standard deviation).

Parameters	G1 pre	G1 post	p-value	G1 post	G2	p-value
SBP (mmHg)	124.5±18	117.3±13	0.001^b^	117.3±13	123.2±1	0.2
DBP (mmHg)	75.8±14	71.9±12	0.006^b^	71.9±12	73.8±6	0.6
Abdominal (cm)	92.5±17	90.6±1	0.03^a^	90.6±15	95.12±14	0.4
BMI (kg/m^2^)	26.7±5	26.3±4	0.25	26.3±4	26.8±4	0.9
Thigh Measure	3.9±1.8	2±1.2	0.003^b^	2±1.2	3.9±1.2	0.001^b^

SBP, Systolic Blood Pressure; DBP, Diastolic Blood Pressure; Abdominal, Abdominal circumference; BMI, Body Mass Index; Thigh Measure, Thigh circumference difference between non-amputation and amputation side.

a p ≤ 0.05.

b p ≤ 0.01; *t-*student and Wilcoxon Sign Rank test (G1 pre/post)

However, after Concurrent Training (CT) the SBP was significantly reduced by 5.6% and 5.1%, respectively in G1 and was lower than in G2 by 5%. There was no difference in DBP in relation to G2 (Table 2). Waist circumference decreased by 2% before and after CT and when compared to G2 it was also lower by 5% (Table 2). BMI was not modified after CT in both groups, respectively (Table 2). The thigh measure was reduced in G1 by 49% after CT and 49% lower than in G2 (Table 2).

The isokinetic performance of G1 before and after CT when compared to the values of G1 in relation to Paralympic athletes are described in Table 3.

**Table 3:** Comparison of the Isokinetic Dynamometry (speed 60°/s) at Group 1 (G1) Unilateral Transtibial Amputation (UTA) patients and Group 2 (G2) – Unilateral Transtibial Amputation (UTA) paralympic athletes (mean and standard deviation).

Variables	G1 pre	G1 post	p-value	G1 post	G2	p-value
**Extension 60^o^/s**						
TWAS	161.2±116	317.8±150	0.001^b^	317.8±150	311.3±180	0.9
TWNAS	545.5±196	582.9±177	0.04^a^	582.9±177	734.4±169	0.02^a^
Delta	71±17	45.8±18	0.0005^b^	45.8±18	58.5±19	0.06
PTCAS	74.5±44	153.5±66	0.0003^b^	153.5±66	137.2±66	0.48
PTCNAS	234.4±55.5	249.4±77	0.04^a^	249.4±77	281.6±46	0.15
**Flexion 60^o^/s**						
TWAS	92.8±55	191.4±86	0.002^b^	191.4±86	198.6±111	0.83
TWNAS	251.6±102	304.3±110	0.001^b^	304.3±110	392.7±106	0.02^a^
Delta	58.6±23	34.9±21	0.0003^b^	34.9±21	51±21	0.03^a^
PTCAS	51.2±26.7	98.9±35.5	0.0003^b^	98.9±35.5	76.5±3	0.06
PTCNAS	102.5±26	127.9±26	0.001^b^	127.9±26	139±33	0.26

TWAS, Total Work Amputation Side; TWNAS, Total Work Non-Amputation Side; Delta, Total work difference between non-amputation and amputation side; PTCAS, Peak Torque Corrected by body weight Amputation Side; PTCNAS, Peak Torque Corrected by body weight Non-Amputation Side.

a p ≤ 0.05.

b p ≤ 0.01; *t*-student and Wilcoxon Sign Rank test (G1 pre/post).

The muscle extensor strength of the amputated side (TWAS) of G1 after CT significantly increased by 97%, but when compared to G2 there was no significant difference (Table 3). G1′s TWNAS increased by 7%, but when compared to G2 it was 21% lower (Table 3). When the authors compared TWAS in extension and flexion of G1 vs. G2, respectively, there was no significant difference (Table 3). G2 had lower results, both in the quadriceps and in the hamstrings, on the amputated side when the authors analyzed the peak torque/weight at 60°/s (a very important variable in the analysis of a patient's strength). G1 had a highly significant gain in extensor delta of 57%. Post G1 had a significant advantage in extensor delta over G2 of 28% (Table 3). After CT, the results of G1 were comparable to the results of Paralympic athletes (Table 3).

Table 4 shows that the cardiorespiratory performance data of G1 were mostly satisfactory after CT and when compared to G2 there was no statistical difference in most variables. The CT over G1 was effective in equalizing the two groups.

**Table 4:** Comparison of the cardiopulmonary exercise test at Group 1 (G1) Unilateral Transtibial Amputation (UTA) patients and at Group 2 (G2) – Unilateral Transtibial Amputation (UTA) paralympic athletes (mean and standard deviation).

Parameters	G1 pre	G1 post	p-value	G1 post	G2	p-value
PeakVO_2_	25.3±6.0	30.9±7.0	0.0007^b^	30.9±7.0	31.8±5	0.71
Peak HR	178.9±13.7	187.6±10.4	0.02^a^	187.6±10.4	183.9±12	0.34
Peak Workload	139.1±40.8	179.4±33.3	0.002^b^	179.4±33.3	204.4±40	0.06
Peak PO_2_	10.4±3.2	12.5±3.4	0.0006^b^	12.5±3.4	13.9±3	0.21
TT	10.2±3.1	13.2±2.8	0.003^b^	13.2±2.8	15.7±3	0.02^a^
%PMHRP	95.0±5.9	100.8±5.3	0.004^b^	100.8±5.3	99.2±6	0.43
%AbVO_2_peak	72.9±13.6	87.4±15	0.005^b^	87.4±15	89.5±13	0.67
ME	12.4±6	11.6±8	0.72	11.6±8	11.5±5	0.96

VO_2_peak, Peak Oxygen uptake at maximal effort, in mL/kg/min; HRmax, Maximum Heart rate reached maximum effort; W, Training load, maximum effort, in watts; PO_2_, Pulse of O_2_ at maximum effort; TT, Time Test, in minutes; %PMHRP, Percentage of Maximum Heart Rate Predicted; %AbVO_2_peak, Percentage absolute maximal oxygen; ME, Mechanical Efficiency.

Table 4 shows that the VO_2_peak of G1 increased by 22% after CT, but when compared to G2 (paralympic athletes) there was no statistical difference (p = 0.71). The peak heart rate before and after CT and compared to Paralympic athletes was not different (p = 0.34). The peak load reached before and after CT in G1 was significantly increased by 29% (p = 0.002), but in relation to G2, there was no statistical difference. The oxygen pulse peak was significantly modified by 22% in G1 after CT, but when compared to G2 there was no difference (p = 0.21) (Table 4). The mechanical efficiency when pedaling on the cycle ergometer was not significantly altered after CT (p = 0.72) nor between G1 and G2 (0.96).

## Discussion

This study demonstrated the feasibility and effectiveness of a resistance and aerobic exercise training program for individuals with amputations. The authors compared the efficiency of supervised exercise with the results of Paralympic athletes. It is important to remember that higher aerobic capacity and greater skeletal muscle strength are associated with a lower prevalence of chronic diseases and lower mortality.^17^

The remarkable increases in max aerobic capacity (22%), oxygen pulse (20%), peak workload in the end exercise testing as well as muscle strength (peak torque corrected by body weight) and endurance (total work amputation) for the lower body (106% and 97%, respectively), demonstrated in this study the aerobic potential of the training program to reduce risks from chronic diseases such as heart disease, stroke, and diabetes, respectively. Nolan^18^ evaluated cardiorespiratory capacity in these patients after aerobic training and found favorable effects that were compatible with the results of the present study. Another aspect that seems important is the intensity of the exercises. A study demonstrated that low-intensity exercise has no training effect in these patients.^5^ The authors believe that the effectiveness of concurrent training applied to these patients had the merit of respecting the FITT principle, that is, intensity, and duration of exercise, type, and frequency of exercise. Intermittent exercise (low intensity at VT_1_) and (high-intensity at VT_2_) performed by the patients seems to have been the riddle of this program.

Another physiological element derived from the improvement of aerobic metabolism was the increase in the Oxygen Pulse (PO_2_). The PO_2_ is one of the important cardiorespiratory variables used for fitness classification. It can be estimated by the ratio of oxygen uptake (VO_2_) and Heart Rate (HR). It is also equal to the product of stroke volume and Arteriovenous Oxygen (A-VO_2_) difference. The authors did not measure the A-VO_2_ difference and cardiac output, but the authors did estimate the O_2_ pulse by measuring VO_2_ and HR. The results of the present study corroborate studies that have shown that unilateral training is effective in increasing PO_2_ caused by the CT effect.^19^, ^20^, ^21^, ^22^ This variable is one of the important cardiorespiratory elements used for fitness classification. The present study did not find any research showing the importance of this parameter in the transtibial amputee. However, the authors understand, increased A-VO_2_ difference is the major contributing factor for increased VO_2_ max and increased exercise tolerance, and this suggests that the change in VO_2_ uptake can directly affect the PO_2_ and A-VO_2_ difference reducing the cardiovascular central energy expenditure of these patients.^23^

It is important to point out that there is research that supports the fact that the higher the amputation level, the energy demand increases, and, therefore, they require considerably higher levels of effort to perform the same rates of work compared to non-individuals amputees.^24^ Therefore, activities that range from low to high exercise intensity, such as the one in the present study, become a great option to optimize energy expenditure in these patients.^25^ Kent et al.^26^ state that the daily use of the prosthesis is sufficient to improve the physical conditioning of the participants. However, this assertion was not confirmed in the present study. Aerobic interval exercises are known to improve VO_2_peak.^27^ The present results are similar to those found by other studies^27^^,^^28^ that demonstrated that this training modality (2 to 15 weeks) increases VO_2_peak between 4% to 46%. These findings increased aerobic power by 22% and were similar to findings in the literature.^27^^,^^28^

Therefore, the main outcome of the study was that the level of strength and cardiorespiratory fitness of the dependent variables increased significantly in G1 patients with Unilateral Transtibial Amputation (UTA) after supervised concurrent training.^29^ However, compared to the “gold standard” group of Paralympic athletes, after the intervention, the authors did not observe a significant difference in several dependent variables. However, the efficiency of CT in G1 was evident, which improved muscle strength and aerobic capacity significantly (*d* – significant Cohen), even starting from a reduced functional capacity and confirmed by other authors.^1^ When the authors compared the main parameters of strength and aerobic power after the concurrent training, the authors did not verify a significant difference with the results of the parathletes, who could be considered as the “gold” standard of physical condition because they are high-performance athletes and in the preparation phase for the Games 2016 Paralympics. The control group made up of parathletes maintained regular levels of activity.

The specific parameters of muscle strength in the pre-training phase of G1 were below what is necessary for the proper use of the prosthesis, since the normal physiological limit and the metabolic cost of gait in TA patients is ten to 30% higher than in non-patients amputees, with 22% less speed and with a high risk of developing cardiovascular overload and chronic diseases, evidencing the need for specific training for the use of the prosthesis and gait.^2^ Several authors^29^^,^^30^ are very clear when they report in their work that the balance of muscle strength between the hamstrings (weaker) and quadriceps (stronger) is essential to avoid injury and improve walking or running performance. In the present study's patients, the authors verified an increase of 97% in strength in the hamstrings of the stump remaining in the ATU, corroborating the value of this physical valence in the rehabilitation of these patients. Pedrinelli et al.^29^ in their work explain that weak knee flexors make it difficult to load the prosthesis in TA and Brugaru et al.^24^ and Schmitt et al.° state that strong flexors prevent muscle and joint injuries in the knee. In another noteworthy testimony, Moirenfeld et al.^31^ comments in their research that, from the metabolic point of view, the muscles of the amputated limb work properly. It is of great importance to reduce the bilateral deficit and the degree of atrophy as soon as possible in order to improve the level of physical performance. By choosing a correct strength and endurance training program, one can expect a significant and good reaction from the muscles of the amputated limb, as is expected from training the muscles of a healthy (non-amputated) limb. The CT of this study confirms the results found by Moirenfeld et al.^31^

The AT muscle condition was assessed through the thigh circumference and isokinetic dynamometry of the knee flexors and extensors. Both groups had muscle deficiency at baseline. Muscular hypotrophy was present: more than 3 cm of difference between the amputated and non-amputated thighs and the maximum extensor torque (velocity of 60°/s) was enough to support only half of the patient's body mass, which means that they were not able to support your body weight on the amputated leg (single leg support). Pedrinelli et al.^29^ found these same perimetry findings and great weakness in the quadriceps and hamstrings when evaluating transtibial amputees with isokinetic dynamometry. The deficiency of the muscular condition is related to the limitation of getting up and walking. Weakness of the knee flexor and extensor muscles impairs the suspension and mobility of the prosthesis, as well as speed, cadence, and step size, increasing energy expenditure during gait.^29^ Ihmes et al.^32^ and Grecco et al.^33^ reports in their research that a simple program to strengthen the stump and the contralateral limb in unilateral transtibial amputees improves speed, balance and suspension of the prosthesis during gait. The perimetry of pre-G1 was the same as that of G2 (the difference between the lower limbs was 3.9 cm). After training, the difference increased to 2 cm in G1. Therefore, the result of the present study confirms the findings of Ihmes et al.^32^ and Grecco et al.^33^

Muscular hypotrophy is related to disuse (surgery and physical inactivity); local pain (stump condition); proprioceptive losses and senescence (20% muscle wasting between 25‒60 years of age).^34^ The hamstring muscles are the most affected by hypotrophy in TA, mainly due to the decreased range of knee flexion during gait. Pedrinelli et al.^16^ state that the changes caused by disuse are similar to physiological aging. All parameters of the flexor muscles had significant gains in G1. The statistical difference is evident from G1 pre to G1 post (Table 3). The flexor delta of the G1 post was statistically superior to G2. Although the peak torque corrected by body weight in the flexors of the amputated side did not give an expressive statistical value, it showed a great evolution of strength. Fisher et al.^35^ and Ihmes et al.^32^ demonstrated that minimum doses of strength exercise per week can make a big difference in the health of frail patients and that even statistically it has not given expressive differences, great functional gait gains are already observed. Schmitt et al.^30^ and Moirenfeld et al.^32^ also support these functional findings and confirm the aspect of psychological improvement (self-esteem). This point was not evaluated but observed during the study.

Muscle strength is considered one of the most important physical fitness qualities for human independence and is critical for amputees.^36^^,^^37^ All results observed in the present study are in agreement with the literature that advocates that muscle strength gain is the driver of significant changes in people with a fragile profile.^27^^,^^31^^,^^34^ The results show that a short-duration program with moderate loads (80% of 1RM), three times a week, promoted a significant improvement in strength and muscular endurance in G1 and are in agreement with results found in the literature.^27^^,^^31^ The authors found a very large effect (*d*-1.30) at peak of absolute torque, peak torque corrected by body weight, and in the delta in the quadriceps of the amputated side. In the hamstring on the amputated side, the authors observed a large effect (*d*-0.80‒1.29) on absolute peak torque and weight-corrected peak torque parameters. Studies have shown that strength training improves isokinetic parameters at 60°/s in the AT.^31^^,^^38^ This is an important aspect, as the improvement in quadriceps muscle mass implies a better performance of the lower limbs during the sit and stand movement, improving the distribution of loads between the lower limbs in these individuals.^39^

Therefore, it is evident from these findings that well-programmed strength training is important in the rehabilitation of fragile groups right after the fitting, as verified in the literature.^34^ Some authors^18^^,^^34^ demonstrated that conventional physiotherapy is not superior to strength training for this group, peremptorily confirming the present results. Patients who were discharged after physiotherapy and prosthesis had muscle weakness and a lack of resistance to perform an efficient gait. The concurrent training design developed by the present study, with only 24 sessions, reversed the physical fragility of the patients. The dependent variable strength, in such a short time, was favorably modified as verified by several authors, increasing the protection of patients.^5^^,^^35^, ^36^, ^37^ The authors can state with the present study's results added to the results of other authors^36^^,^^37^ that strength work is essential for amputees. CT encompasses the need of combining cardiorespiratory endurance and muscular strength in amputees. This position is true and these results are in agreement with several studies.^24^^,^^40^^,^^41^

It is not new that the literature confirms the favorable effect of aerobic exercise on blood pressure.^25^ Blood pressure, waist circumference, and difference in measurements between the two thighs decreased after eight weeks of CT, showing positive adaptation in trained amputees. When comparing G1 after CT with the parameters of G2, in the clinical evaluation, the authors did not observe any statistical difference. The G1 of sedentary amputees showed better values in all clinical parameters analyzed after CT. The BMI was not modified in G1, but there was a change in body composition with an increase in muscle mass and a reduction in localized fat, and this theory was confirmed when the authors analyzed the significant statistic in the abdominal circumference. Out of curiosity, the effect size of the thigh difference had Cohen's *d* = 1.17 (large effect). Examining Table 2, in addition to the observation of a great evolution of G1, the authors noticed that this group when compared to G2 (parathletes) in several variables did not present a statistically significant difference, which was already expected (Tables 2, 3 and 4). It is undeniable that even without major statistical differences between groups, CT training is essential to change the clinical state of UTA. Gains in muscle mass, decrease in body fat and, greater efficiency in the cardiorespiratory system functionally benefit walking amputees and any other group that is weakened in their general condition. These findings and statements are found in the works of several authors^3^^,^^4^^,^^8^ and only confirm these findings. More important than showing large statistical differences is showing the power of CT training in a clinical and physical change in this group. This study shows this, corroborating with the authors already cited above.

Aerobic interval exercises are known to improve VO_2_peak.^27^ This training modality (2 to 15 weeks) increases VO_2_peak between 4% to 46%.^27^^,^^28^ The present findings increased aerobic power by 22% and were similar to other studies.^28^ However, when compared to G2 (parathletes) there was no significant difference, which confirms previous studies.^28^ The association with resistance exercises contributed to a greater gain in the aerobic condition of the trained group (G1) which was related to a greater gain in muscle mass and confirms that aerobic exercise acutely and chronically alters protein metabolism and induces skeletal muscle hypertrophy.° When the authors look at Table 4, we can see that, except for mechanical efficiency, all other variables in G1 after CT had significant gains with considerable statistical differences. The limitation of the cardiorespiratory system can be central (cardiac output and arterial O_2_ concentration) and/or peripheral (arteriovenous difference and tissue metabolism), causing functional impairment of the locomotor system in AT, with decreased oxygen transport and muscle function.^41^

Kent et al.^26^ state that the daily use of the prosthesis is sufficient to improve the physical conditioning of the participants. However, this assertion was not confirmed in the present study. The average time of amputation of the amputees was 56 months and when they were evaluated, they did not show satisfactory results. The post-TC G1 had an improvement in their VO_2_max of 22%. When comparing post-G1 with G2, the authors did not find any statistical difference, except for the variable of longer duration of effort in the test. The training effect size in G1 was large (*d* = 0.89‒1.29) on aerobic parameters in VO_2_max, W, TD and %PMHRP. The training three-time-a-week was effective, with the same results observed by Carvalho et al.^36^ who showed that CT is efficient in very frail participants. It is also in agreement with the findings of Jarvis et al.^42^ where comprehensive rehabilitation is more efficient to improve gait than just a sophisticated device. Other studies report that eight weeks of strength training are sufficient to cause hypertrophy and functional improvement even without aerobic training.^5^ On the contrary, one study reports that CT impairs muscle strength and hypertrophy.^43^ The authors did not observe this statement in the present study. Unfortunately, mechanical efficiency has not improved, but this is probably due to the high energy cost of type II glycolytic fibers that are increased in this population and, therefore, more fatiguable, to the detriment of type I (aerobic) muscle fibers.^44^

Better Mechanical Efficiency (ME) is linked to a greater economy of movement. Thus, it helps to reduce the oxygen demand to perform movements for longer and for greater distances at a given speed and can generate increases in resistance performance, an important condition for the amputee.^18^^,^^24^

When the authors analyzed this variable (the closer to 25%, the better) together with the others from the cardiopulmonary evaluation, we found that the Paralympic athletes (G2) were not statistically superior to G1. It is clearly noted that the EM is low in both groups of amputees, which characterizes high energy expenditure in these patients.

The results of the present study show that a regular physical conditioning program for the TA after fitting is necessary to achieve adequate functional performance. The same recommendation is valid for parathletes, in the improvement of athletic performance. CT does not require expensive equipment and good results are directly related to the prior assessment of metrics, an efficient rehabilitation program, and progress monitoring, always seeking maximum performance.^5^ Prosthetic gait requires more energy than normal gait and therefore requires strength and power training for greater efficiency.^45^

### Application in rehabilitation services

This study brings an important message to the field of rehabilitation for amputees. Lower limb amputees have less exercise capacity needed to use their well-fitting prosthesis. He showed that a well-controlled training program based on the principles of physical training and without many equipment resources can have consistent effects on the cardiorespiratory and metabolic systems, reducing the cardiovascular load in these individuals.^23^ Therefore, previously evaluating the cardiovascular response of amputees, whether through the lower or upper limbs, is the most appropriate approach to verify the limitations of exercise.^23^ This evaluation will make it possible to adequately and safely prescribe the exercise intensity. Monitoring heart rate response and blood pressure is another important point, as many individuals may have an abnormal response when compared to non-amputees. These individuals may have possible underlying diseases (hypertension, diabetes, heart disease, etc.) and underlying changes.^44^ An important factor, physical training must be personalized in the amputee population, as it is very heterogeneous in terms of disability and capacity (orthopedic, vascular, and cardiac).^25^^,^^46^

### Study limitations

The main limitation of the present study was the sample size, the G2 group (parathletes) did not have the same training and control as the G1. The socioeconomic level of G1 was low, making it difficult to travel to the hospital, the availability of time to carry out the training program and the clinical implications made it difficult to include a greater number of patients. Therefore, the comparison with the present study is diversified, making a more robust synthesis difficult. The possibility of type II error due to the variability of data within a relatively small sample must be acknowledged, particularly with regard to outcomes. Another important issue is that the lack of long-term follow-up prevents a better analysis of the benefits of the methods used.

## Conclusion

The increase in dependent variables in patients with ED after an 8-week supervised concurrent training program is indicated for patients with unilateral transtibial amputation and met the expectations of improving muscle strength and cardiorespiratory fitness due to better adaptations in the variables central and peripheral. Rehabilitation centers, whether conventional or sports, specializing in amputees, should rethink their approaches to AT, putting a simple and effective method, such as concurrent training, to improve performance, in the case of parathletes, and faster and more efficient treatment of non-athlete amputees. The relevance of the results in the present study indicates that aerobic interval training and resistance training are the most suitable for this population because they provide significant improvements in strength, balance, autonomy, functional performance, physiological performance, and, above all, decreasing energy expenditure, in addition to being associated with favorable changes in cardiorespiratory function.

## CRediT authorship contribution statement

**Marcus Vinicius Grecco:** Investigation, Writing – review & editing. **Guilherme Carlos Brech:** Investigation, Writing – review & editing. **Jose Maria Soares-Junior:** Investigation, Writing – review & editing. **Edmund Chada Baracat:** Investigation, Writing – review & editing. **Júlia Maria D'Andrea Greve:** Supervision, Writing – review & editing. **Paulo Roberto Santos Silva:** Investigation, Writing – review & editing.

## Conflicts of Interest

The authors declare no conflicts of interest.

## References

[1] Pastre CM, Salioni JF, Oliveira BA, Bruno AF, Micheletto M, Netto J (2005). Fisioterapia e amputação transtibial Physical therapy and transtibial amputation. Arq Ciênc Saúde.

[2] van Velzen JM, van Bennekom CAM, Polomski W, Slootman JR, Woude LHV, Houdijk H. (2006). Physical capacity and walking ability after lower limb amputation: A systematic review. Clin Rehabil.

[3] Bussmann JB, Schrauwen HJ, Stam HJ. (2008). Daily physical activity and heart rate response in people with a unilateral traumatic transtibial amputation. Arch Phys Med Rehabil.

[4] Christiansen C, Fields T, Lev G, Stephenson RO (2015). Stevens-Lapsley JE. Functional outcomes following the prosthetic training phase of rehabilitation after dysvascular lower extremity amputation. PMR.

[5] Wezenberg D, van der Woude L, Faber W, de Haan A, Houdijk H. (2013). Relation between aerobic capacity and walking ability in older adults with a lower-limb amputation. Arch Phys Med Rehabil.

[6] Fernandez-Gonzalo R, Lundberg TR, Tesch PA. (2013). Acute molecular responses in untrained and trained muscle subjected to aerobic and resistance exercise training versus resistance training alone. Acta Physiol.

[7] Gailey RS, Wenger MA, Raya M, Kirk N, Erbs K, Spyropoulos P (1994). Energy expenditure of trans-tibia1 amputees during ambulation at self-selected pace. Prosthet Orthot Int.

[8] Mohanty RK, Lenka P, Equebal A, Kuma R (2012). Comparison of energy cost in transtibial amputees using “prosthesis”and “crutches without prosthesis” for walking activities. Ann Phys Rehabil Med.

[9] Tsitkanou S, Spengos K, Stasinaki AN, Zaras N, Bogdanis G, Papadimas G (2017). Effects of high-intensity interval cycling performed after resistance training on muscle strength and hypertrophy. Scand J Med Sci Sports.

[10] Methenitis S. (2018). A brief review on concurrent training: from laboratory to the field. Sports.

[11] Hijmans JM, Dekker R, Geertzen JHB. (2020). Pre-operative rehabilitation in patients with lower limb amputations and its effect on postoperative outcomes. Medical Hypotheses.

[12] Williams B, Mancia G, Spiering W, Dominiczak A, Kahan T, Mahfoud F (2018). 2018 ESC/ESH guidelines for the management of arterial hypertension: the Task Force for the Management of Arterial Hypertension of the European Society of Cardiology and the European Society of Hypertension. J Hypertens.

[13] Salguero GC, Gosalvez AP, Rebollo JMC, Fernandez IB, Rosa LF, San Jose FGM. (2021). Isokinetic profiles and reference values of professional soccer players. Rev Bras Med Esporte.

[14] Hottenrot K, Ludyga S, Schulze S. (2012). Effects of high intensity training and continuous endurance training on aerobic capacity and body composition in recreationally active runners. J Sports Sci Med.

[15] Baechle T, Earle R, Wathen D, Baechle T., Earle R. (2008). Essentials of Strength and Conditioning.

[16] Haskell WL, Lee IM, Pate RR, Thompson PD, Bauman A, Blair SS. (2007). Physical activity, and public health: updated recommendation for adults from the American College of Sports Medicine and the American Heart Association. Med Sci Sports Exerc.

[17] Booth FW, Roberts CK. (2008). Linking performance and chronic disease risk: indices of physical performance are surrogates for health. Br J Sports Med.

[18] Nolan L. (2012). A training programme to improve hip strength in persons with lower limb amputation. J Rehabil Med.

[19] Carroll TJ, Herbert RD, Munn J, Munn J, Lee M, Gandevia SC. (2006). Contralateral effects of unilateral strength training: evidence and possible mechanisms. J Appl Physiol.

[20] Adamson M, Macquaide N, Helgerud J, Hoff J, Kemi OJ. (2008). Unilateral arm strength training improves contralateral peak force and rate of force development. Eur J Appl Physiol.

[21] Lee M, Gandevia SC, Carroll TJ. (2009). Unilateral strength training increases voluntary activation of the opposite untrained limb. Clin Neurophysiol.

[22] Song Y, Forsgren S, Yu J, Lorentzon R, Stål PS (2012). Effects on contralateral muscles after unilateral electrical muscle stimulation and exercise. PLoS ONE.

[23] Haddad S., Ergometria de membros superiores (1997). Um método importante na avaliação cardiocirculatória ao exercício. Arq Bras Cardiol.

[24] Brugaru M, Dekker R, Geertzen JHB, Dijkstra PU. (2011). Amputees and Sports – A Systematic Review. Sports Med.

[25] Bosser G, Martinet N, Rumilly E, Paysant J, André J-M. (2008). Exercise training for lower limb amputees. Ann Readapt Med Phys.

[26] Kent JA, Stergiou N, Wurdeman SR. (2017). Dynamic balance changes within three weeks of fitting a new prosthetic foot component. Gait Posture.

[27] Boutcher SH. (2011). High-intensity intermittent exercise and fat loss. J Obes.

[28] Kessler HS, Sisson SB, Short KR. (2012). The potential for high-intensity interval training to reduce cardiometabolic disease risk. Sport Med.

[29] Pedrinelli A, Saito M, Coelho RF. (2002). Comparative study of the strength of the flexor and extensor muscles of the knee through isokinetic evaluation in normal subjects and patients subjected to trans-tibial amputation. Prosthet Orthot Int.

[30] Schmitt B, Tim T, McHugh M. (2012). Hamstring injury rehabilitation and prevention of reinjury using lengthened state eccentric training: a new concept. Int J Sports Phys Ther.

[31] Moirenfeld I, Ayalon M, Ben-Sira D. (2000). Isokinetic strength and endurance of the knee extensors and flexors in trans-tibial amputees. Prosthet Orthot Int.

[32] Ihmes WD, Miller RH, Esposito ER. (2022). Residual limb strength and functional performance measures in individuals with unilateral transtibial amputation. Gait Posture.

[33] Grecco MV, Brech GC, Camargo CP, Santos-Silva PR, Greve JMDA. (2023). The eight-week concurrent training effect on functional capacity in person living with unilateral transtibial amputation: a randomized controlled trial. J Bodywork Movement Therap.

[34] Schafer ZA, Perry JL, Vanicek N. (2018). A personalised exercise programme for individuals with lower limb amputation reduces falls and improves gait biomechanics: A block randomised controlled trial. Gait Posture.

[35] Fisher JP, Steele J, Gentil P. (2017). A minimal dose approach to resistance training for the older adult; the prophylactic for aging. Exp Gerontol.

[36] Carvalho LP, Pion CH, El Hajj Boutros G, Gaudreau P, Chevalier S, Belanger M (2019). Effect of a 12-week mixed power training on physical function in dynapenic-obese older men: does severity of dynapenia matter?. Aging Clin Exp Res.

[37] Yoon DH, Lee JY, Song W. (2018). Effects of resistance exercise training on cognitive function and physical performance in cognitive frailty: a randomized controlled trial. J Nutr Heal Aging.

[38] Gentil P, Del Vecchio FB, Paoli A, Schoenfeld BJ, Bottaro M (2017). Isokinetic dynamometry and 1RM tests produce conflicting results for assessing alterations in muscle strength. J Hum Kinet.

[39] Nardone A, Schieppati M. (2010). The role of instrumental assessment of balance in clinical decision making. Eur J Phys Rehabil Med.

[40] Konopka AR, Harber MP. (2014). Skeletal muscle hypertrophy after aerobic exercise training. Exerc Sport Sci Rev.

[41] Timmons JF, Minnock D, Hone M, Cogan KE, Murphy JC, Egan B. (2018). Comparison of time-matched aerobic, resistance, or concurrent exercise training in older adults. Scand J Med Sci Sport.

[42] Jarvis HL, Bennett AN, Twiste M, Phillip RD, Etherington J, Baker R (2017). Temporal spatial and metabolic measures of walking in highly functional individuals with lower limb amputations. Arch Phys Med Rehabil.

[43] Coffey VG HJ (2017). Concurrent exercise training: do opposites distract?. J Physiol.

[44] Lacour JR. (2011). Muscle activity and energy expenditure. Rev Mal Respir.

[45] Chamlian TR, Angrisani PG, Resende JM, Celestino ML, Gramani K, Barela AMF. (2013). Avaliação do padrão postural e marcha de pacientes amputados vasculares transtibiais protetizados. Acta Fisiátrica.

[46] Kodama S, Saito K, Tanaka S, Maki M, Yachi Y, Asumi M (2009). Cardiorespiratory fitness as a quantitative predictor of all-cause mortality and cardiovascular events in healthy men and women: a meta-analysis. JAMA.

